# Defining new SNARE functions: the i-SNARE

**DOI:** 10.3389/fpls.2013.00099

**Published:** 2013-04-16

**Authors:** Gian-Pietro Di Sansebastiano

**Affiliations:** Laboratory of Botany, DiSTeBA, University of SalentoLecce, Italy

SNAREs (N-ethylmaleimide-sensitive factor adaptor protein receptors) have been often seen to have a dishomogeneous distribution on membranes and are apparently present in excess of the amount required to assure correct vesicle traffic. It was also shown in few cases that SNARE on the target membrane (t-SNARE) with a fusogenic role, can become non-fusogenic when overexpressed.

When SNAREs concentration is inversely proportional to the expected fusogenic activity, they can be reasonably defined as “inhibitory” or “interfering” (i-SNAREs). In fact i-SNAREs have been proposed to form a new functional class of SNAREs. In this manuscript I discuss data obtained in various eukaryotic models that leave open different possibilities for the action mechanism of the i-SNAREs in plants.

## SNARE abundance and influence of distribution on their fusogenic role

SNAREs are relatively small polypeptides (~200–400-amino-acids) characterized by the presence of a particular domain, the SNARE motif (Jahn and Scheller, 2006), consisting of heptad repeats that can form a coiled-coil structure. Via hetero-oligomeric interactions, these proteins form highly stable protein-protein interactions organized in a SNARE-complex that help to overcome the energy barrier required for membrane fusion. SNAREs also interact with several proteins acting as regulators of SNARE-complex formation (Lobingier and Merz, 2012; Schafer et al., 2012).

Even after considering all these potential interactors, in living cells, most SNARE molecules are apparently present in excess and concentrated in clusters, thus constituting a spare pool not readily available for interactions.

This consideration was first formulated by Bethani and co-workers upon analysis of a rich literature showing that knockdown of some SNARE genes have little effect on endomembranes (Bethani et al., 2009, and references within). It was shown that in the cells where SNAREs were silenced using siRNA, membrane compartments exhibited an enhanced docking instead of the expected inhibition. In particular the authors observed that there were proportionally more docked endosomes in the syntaxin 13 knockdown, compared with the control. About the alteration of SNARE function, it is essential to remember that antibodies or recombinant SNARE fragments, showing inhibitory or dominant negative (DN) effect, for example, on syntaxin 13 (Bethani et al., 2009), induce effects that are very different: antibodies cause the depletion of active domains while SNARE fragments cause the competitive saturation of the interacting partners. The reduction of gene expression by siRNA, when other homologous gene products remain expressed, has a very different effect too.

SNAREs (precisely t-SNAREs) have been visualized to form apparent clusters using fluorescence and confocal microscopy. This inhomogeneous distribution was initially proposed to provide a localized pool of t-SNAREs to facilitate and enhance membranes fusion (van den Bogaart et al., 2011) but recently, using super-resolution microscopy techniques, Yang and coworkers (2012) showed that secretory vesicles were preferentially targeted to membrane areas with a low density of SNAREs. This last study was surprising because it showed that a higher concentration of SNAREs was not correlated to more frequent fusion events. It supported the idea that a small number of SNAREs is needed to drive a single fusion event and that the proteins not engaged in classic fusion events are maintained, by yet undefined mechanisms, in membrane micro-domains with a non-random molecular composition. Vesicles do not preferentially target these microdomains.

Several mechanisms have been proposed to explain protein clustering in micro-domains and the t-SNARE distribution seems to depend both on lipidic and proteic contributions (Yang et al., 2012). Regulating t-SNARE distribution the cell could dynamically modulate vesicle fusion probabilities and consequently the kinetics of the cellular response (Silva et al., 2010; Yang et al., 2012). Additional structural roles for t-SNAREs cannot be excluded.

Recently we observed for Arabidopsis SYP51 and SYP52 a double localization associated to two different functions (De Benedictis et al., 2012). The work was almost entirely performed in protoplast with large use of transient transformation but clearly showed that fusogenic and non-fusogenic functions can be ascribed to the same SNARE and be dependent on protein localization. When anchored to the TGN membrane, AtSYP51, and AtSYP52 behaved as t-SNARE, with a fusogenic role, but when they were sorted to the tonoplast their role become non-fusogenic. Despite a certain level of functional specificity, they both seemed to play a structural role in the tonoplast formation by influencing the arrival of new membrane from pre-vacuolar compartments. Also in *Petunia hybrida*, the single SYP51 gene cloned up to now (Faraco, 2011) seems to define in petal epidermal cells a very well defined vacuolar compartments separated from the central vacuole and already observed with other vacuolar markers (Verweij et al., 2008).

The discovery of new structural roles for SNAREs, eventually related to the interaction with still unknown partners, may shed light on vacuolar complex organization and it is not surprising that results about vacuolar SNAREs still appear contradictory.

The vacuolar Qa-SNAREs of SYP2 family where recently judged totally redundant in function (Shirakawa et al., 2010) but their partner-SNAREs, the Qb-SNAREs Vti11, and Vti12 (Sanmartin et al., 2007) and Qc-SNAREs SYP51 and SYP52 (De Benedictis et al., 2012) were found functionally different. More contradictory work was published about SYP2s. Tyrrell and co-workers (Tyrrell et al., 2007) found that the soluble DN variant of SYP21 in *Arabidopsis thaliana* blocks traffic of TIP1:1-YFP chimera to the tonoplast but, it has been also demonstrated that over-expression of SYP21 inhibited PVC-to-vacuole traffic *in vivo* (Foresti et al., 2006).

The definition of redundancy or specificity of plant SNAREs function will probably generate further contradiction due to the experimental approach. The experience matured in our studies on SNARE function specificity tells us that a recognized functional redundancy of SNARE and their abundance in the endomembrane system might prove an obstacle in using stable transformation and knockout plants for this kind of studies. Overexpression of DN variants to inhibit specific functions seems to offer a better tool to affect the molecular machinery leaving no options to the plant cell to adapt and mask the effect (Rehman et al., 2008; Silva et al., 2010; De Caroli et al., 2011; De Benedictis et al., 2012).

Bethani and co-workers (2009) discussed interesting points proving SNARE specificity. They concluded that SNAREs are expressed at much higher levels than needed for maintenance of compartments fusion and that loss of SNAREs is compensated by the co-regulation of the docking machinery.

Little attention is generally paid to the need of the cell to keep very similar compartments separated, because this need may not be evident among endosomes as much as among larger vacuolar structures typical of only few plant cells (Epimashko et al., 2004; Verweij et al., 2008). Proteolipidic composition appears determinant (Strasser et al., 2011).

From new data about vacuolar fusion in yeast, it seems that different SNAREs actively bind to different V-ATPase subunits, influencing their interaction with the proteolipid cylinder so promoting, or inhibiting, the lipid reorientation for the formation of a lipidic fusion pore (Strasser et al., 2011). It is extremely interesting a recent report on SNAREs interaction with proteolipid (Di Giovanni et al., 2010). It was suggested that this interaction had the effect to concentrate SNAREs in some areas to enhance their fusogenic potential but it is now evident that more regulatory events than simple localization is involved. The possibility of a direct interaction between dissociated SNAREs and other transmembrane proteins influencing the lipid bilayer is extremely interesting and may represent a further level of specific regulation of membrane traffic.

## i-SNAREs

At the moment, in plants, it was observed that SYP21 (Foresti et al., 2006), SYP51, and SYP52 (De Benedictis et al., 2012) inhibit vacuolar traffic when overexpressed. Waiting for new data about possible interactions of SYP2s and SYP5s with partners influencing membrane potential or lipid reorientation, we have to better characterize their unexpected interfering effect on vacuolar targeting. When their concentration is inversely proportional to the expected fusogenic activity, SNAREs can be reasonably defined as i-SNAREs. These have been proposed to belong to a new functional class of SNAREs (Varlamov et al., 2004). The proposed model is that i-SNAREs inhibit fusion by substituting for or binding to a subunit of a fusogenic SNARE pin to form a nonfusogenic complex, this hypothesis being confirmed on Golgi-localized SNAREs.

Varlamov and co-workers (2004) suggested that non-fusogenic SNARE complexes, including the i-SNARE partners, have the physiological function at the level of the Golgi apparatus to increase the polarity of this organelle. This would ensure that ER-derived vesicles fuse with the cis-Golgi, while retrograde transport vesicles from endosomes fuse to the trans-Golgi.

Mammalian and yeast i-SNAREs (syntaxin 6/Tlg1, GS15/Sft1, and rBet1/Bet1) were found functionally conserved but i-SNARE characterization in plants is still poor. The recent investigation of SYP5s i-SNARE effect (De Benedictis et al., 2012) may help to discover the molecular mechanisms of this process.

A mechanism for the i-SNARE effect of yeast Qc-SNAREs is described by the competition between endosomal (Tlg1 and Syn8) and vacuolar form (Vam7) of the proteins (Izawa et al., 2012) and because of their ability to interact with V-ATPase subunits influencing membrane potential (Strasser et al., 2011). More proteins potentially able to interact with SNAREs can have a direct influence on membrane potential such as ion channels, as shown in the case of SYP121, able to interact and control the K(+) channel KC1 (Grefen et al., 2010).

The speculations about the mechanism active in plant cells can include the mechanisms elucidated in yeast cells with the exception that in *S. cerevisiae* a single Qc-SNARE is active at each step but more than one are active in plants. Moreover in plant cells the same Qc-SNARE (SYP51/52) was found to be, at the same time, inhibitory when accumulated on the tonoplast but normally fusogenic when located on intermediate compartments (De Benedictis et al., 2012).

A plant characteristic i-SNARE activity could be determinant on vacuoles or post-Golgi endocytic compartments since these compartments appear to diversify between plants and non-plant organisms (Richter et al., 2009). In fact, the understanding of i-SNAREs function may have extreme relevance in the comprehension of vacuolar complex organization since it may regulate homotypic membrane fusion events among large vacuolar compartments.

Several sorting processes may be influenced by the higher concentration of specific SNAREs but the phenomena are simply not yet correlated. As an example we can mention the recent report showing that vacuolar sorting receptors (VSRs), normally targeted to the vacuole, are diverted to the PM in germinating pollen tubes (Wang et al., 2011) where SYP5s are expressed at higher levels than in all other tissues (Lipka et al., 2007; De Benedictis et al., 2012). Considering that overexpression of SYP5s diverted vacuolar markers to the surface of Arabidopsis protoplasts (De Benedictis et al., 2012) a more accurate study may probably correlate in the future the SYPs expression with VSRs sorting to PM.

## Conclusions

SNAREs accumulation outside active complexes may gain a structural role in the maintenance of membrane identity. The equilibrium between fusogenic (t-SNARE) and non fusogenic (i-SNARE) activity of specific SNAREs may reside on their localization, as highlighted for SYP51 and SYP52 (De Benedictis et al., 2012) but also on the formation of “clusters” in cholesterol-containing microdomains (Sieber et al., 2006, 2007).

**Figure 1:**
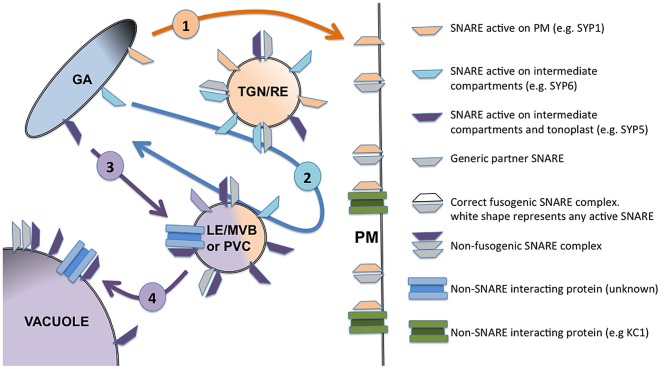
**Schematic representation of four distinct situations described for SNARE functioning in the post-Golgi membrane traffic.** SNAREs active on the PM, such as for example SYP121 and other members of the SYP1 family, are sorted to the target membrane with an inactive conformation where they act as t-SNAREs. They can interact and eventually regulate non-SNARE partners (as shown for SYP121 and KC1 channel) but do not show interfering activity (1). SNAREs can also be specifically localized and active as t-SNARE on intermediate compartments, such as for example SYP61, localized on the TGN membranes (2). Some SNAREs can be distributed on several membranes and change their action mechanism, as it was shown in the case of SYP5s: they can be active as t-SNARE on the TGN and/or PVC (3), or as i-SNARE when accumulated on the tonoplast (4). The compartments indicated in the figure are generic; their identity may change in different experimental systems and in differentiated cells. The figure summarize five membranes belonging to: the Golgi apparatus cisternae (GA), the early endosomes often identified with trans Golgi networks or recycling endosomes (TGN/RE), the late endosome in the form of multivesicular bodies or pre-vacuolar compartment (LE/MVB or PVC), and finally the tonoplast of the vacuole.

SYP5s will act as t-SNARE when present on the membrane of TGN or late endosomes and PVC, whereas they will behave as i-SNARE when accumulating on tonoplast (Figure 1). For SNAREs involved in vacuolar sorting I propose that the accumulation on tonoplast may happen by default, being related to an excess of protein thus representing a sort of negative feedback control system to limit the arrival in the central vacuole of the cargo controlled by, for example, the SYP5 complexes.

The distribution of each SNARE on different membranes allows a diversified interaction with SNARE and non-SNARE partners on each membrane, so inducing diversified correlated effects.
